# An iterative computational design approach to increase the thermal endurance of a mesophilic enzyme

**DOI:** 10.1186/s13068-018-1178-9

**Published:** 2018-07-09

**Authors:** Deanne W. Sammond, Noah Kastelowitz, Bryon S. Donohoe, Markus Alahuhta, Vladimir V. Lunin, Daehwan Chung, Nicholas S. Sarai, Hang Yin, Ashutosh Mittal, Michael E. Himmel, Adam M. Guss, Yannick J. Bomble

**Affiliations:** 1 Biosciences Center, National Renewable Energy Laboratory, 15013 Denver West Parkway, Golden, CO 80401 USA; 2 Department of Chemistry & Biochemistry and the BioFrontiers Institute, University of Colorado, Boulder, CO 80309 USA; 3 Biosciences Division, Oak Ridge National Laboratory, Oak Ridge, TN 37831 USA

**Keywords:** Computational protein design, Biofuels, Thermal stability, Pyruvate decarboxylase

## Abstract

**Background:**

Strategies for maximizing the microbial production of bio-based chemicals and fuels include eliminating branched points to streamline metabolic pathways. While this is often achieved by removing key enzymes, the introduction of nonnative enzymes can provide metabolic shortcuts, bypassing branched points to decrease the production of undesired side-products. Pyruvate decarboxylase (PDC) can provide such a shortcut in industrially promising thermophilic organisms; yet to date, this enzyme has not been found in any thermophilic organism. Incorporating nonnative enzymes into host organisms can be challenging in cases such as this, where the enzyme has evolved in a very different environment from that of the host.

**Results:**

In this study, we use computational protein design to engineer the *Zymomonas mobilis* PDC to resist thermal denaturation at the growth temperature of a thermophilic host. We generate thirteen PDC variants using the Rosetta protein design software. We measure thermal stability of the wild-type PDC and PDC variants using circular dichroism. We then measure and compare enzyme endurance for wild-type PDC with the PDC variants at an elevated temperature of 60 °C (thermal endurance) using differential interference contrast imaging.

**Conclusions:**

We find that increases in melting temperature (*T*_m_) do not directly correlate with increases in thermal endurance at 60 °C. We also do not find evidence that any individual mutation or design approach is the major contributor to the most thermostable PDC variant. Rather, remarkable cooperativity among sixteen thermostabilizing mutations is key to rationally designing a PDC with significantly enhanced thermal endurance. These results suggest a generalizable iterative computational protein design approach to improve thermal stability and endurance of target enzymes.

**Electronic supplementary material:**

The online version of this article (10.1186/s13068-018-1178-9) contains supplementary material, which is available to authorized users.

## Background

Advanced biofuels can meet up to three billion barrels of liquid fuel demand on a yearly basis, but face high costs and process complexity as obstacles to commercial implementation [1, 2]. Consolidated bioprocessing (CBP) offers a way to address both cost and process complexity by combining cellulase production, enzymatic digestion, and fermentation into a one-pot reaction [3]. The challenge of CBP is to engineer a single microbe that has the capability of deconstructing cellulosic biomass and fermenting the released sugars to biofuels or bio-based chemicals at sufficiently low cost. Promising CBP microbes are able to effectively deconstruct plant biomass to elemental sugars [4–6], but still require optimization to increase the fermentation of those sugars to products such as bioethanol [7–9].

Metabolic pathway engineering of CBP microbes has focused on achieving chemical outputs near theoretical yields. Streamlining metabolic pathways by removing enzymes at branched points is a common approach to increase chemical yields. Alternatively, branch points can be bypassed entirely through the introduction of nonnative enzymes. Introducing a nonnative enzyme can be difficult, however, if the enzyme has not evolved to function in an environment similar to that of the host organism. In these cases, the hope is that protein engineering can be used to rationally engineer enzymes, increasing the range of tools available for metabolic pathway engineering.

**Fig. 1:**
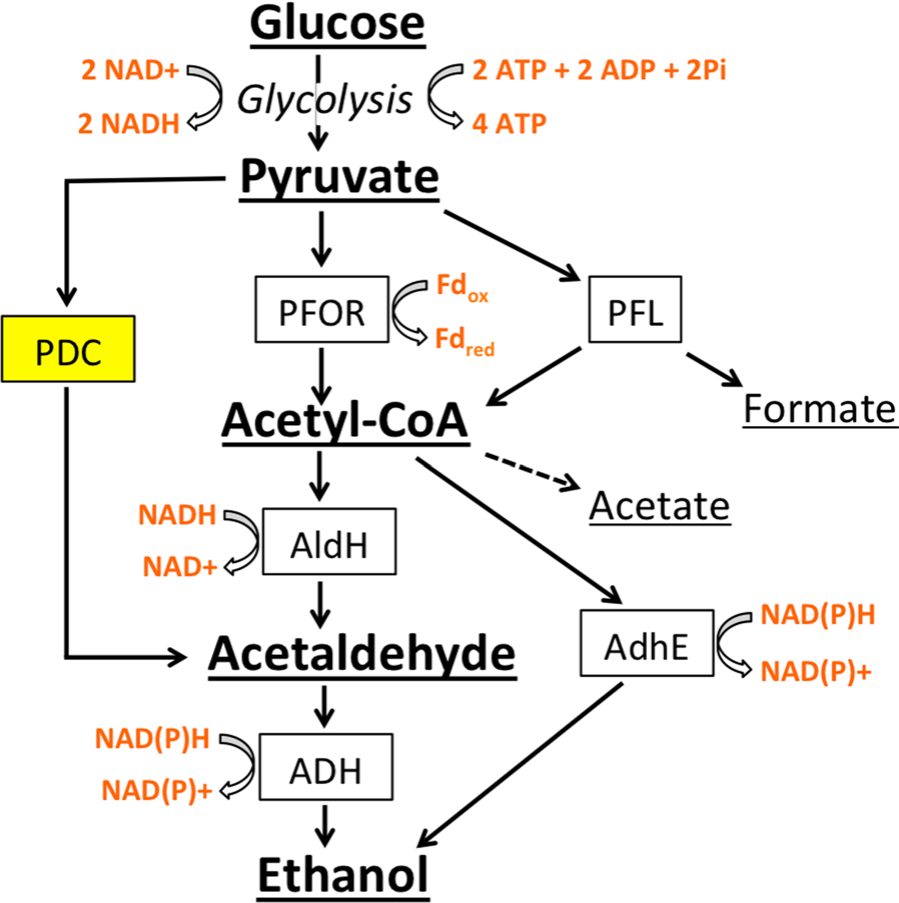
The addition of PDC to fermentative pathways in cellulolytic microbes can add a metabolic shortcut. The pathway shown highlights key steps in *C. thermocellum* fermentation metabolism. The conversion of glucose to pyruvate is summarized as Glycolysis. Enzymes are shown in boxes, redox cofactors in orange, and chemical species are underlined. PDC converts pyruvate directly to acetaldehyde, bypassing acetyl-CoA. Enzyme names are as follows: PFOR (pyruvate:ferredoxin oxidoreductase), PFL (pyruvate formate lyase), AldH (aldehyde dehydrogenase), AdhE (bifunctional aldehyde and alcohol dehydrogenase), and ADH (alcohol dehydrogenase)

Pyruvate decarboxylase (PDC) converts pyruvate to acetaldehyde through nonoxidative decarboxylation, a reaction that typically occurs in anaerobic fermentations. An alternative metabolic route for converting pyruvate to ethanol found in fermentative cellulolytic organisms is branched and produces several additional products including acetate, formate, and lactate [10]. Expression of a PDC in such CBP microbes can bypass these branched points, channeling more pyruvate directly to acetaldehyde for conversion to ethanol (Fig. 1).

Incorporation of the *Z. mobilis* PDC and alcohol dehydrogenase (ADH) into the cellulolytic mesophile, *Clostridium cellulolyticum*, resulted in a 53% increase in the production of ethanol and a 48% decrease in the production of lactate [11]. *Clostridium thermocellum* is a highly cellulolytic thermophile that has emerged as one of the most promising CBP microbes [12]. To date, however, PDC has not been identified in any thermophilic organism, challenging the incorporation of PDC into microbes such as *C. thermocellum*. The potential for using enzymes like PDC to increase yields of renewable fuels and chemicals demonstrates the value of combining computational protein design and metabolic engineering [13].

Protein design tools enhance our ability to manipulate the physicochemical properties of target enzymes. While a majority of randomly selected mutations are deleterious, substitutions identified using computational approaches have a much higher rate of success. In fact, previous computational protein design results demonstrate that multiple independent mutations can be combined to efficiently enhance the thermostability of a target protein without having to screen each substitution individually [14, 15]. Although there are examples of proteins that have been made more thermostable by computational protein design methods, many previous examples have been smaller monomeric proteins [16, 17]. Engineering a larger tetrameric protein like PDC poses additional design challenges. For example, large, multidomain proteins likely have complex folding trajectories and therefor may not have energy minima corresponding to single stable conformations [18]. Thus, larger proteins may require stabilization in more than one structural region in order to observe significant increases in thermal stability.

Engineering an enzyme to function in a thermophilic organism requires increasing the thermal endurance of the enzyme at the growth temperature of the host and may not directly correlate with increases in melting temperature. *C. thermocellum*, for example, grows at 60 °C [19], which is well above the growth temperature of *Z. mobilis* (maximum growth temperature 42 °C) [20] but just below the melting temperature (*T*_m_) of the *Z. mobilis* PDC (62 °C) (Additional file 1: Figure S1A). Changes in ellipticity at 60 °C indicate PDC is within a structurally unstable temperature. When seeking to engineer a mesophilic enzyme for use in a thermophilic host organism it is unknown what the target melting temperature should be to achieve an extended enzyme lifetime, or thermal endurance, at the target temperature.

Here we engineer PDC enzymes with increased thermal stability using the Rosetta protein modeling software [21]. We screen the PDC designs using circular dichroism to identify designs with increased resistance to thermal denaturation. We then monitor the lifespan of the enzymes at 60 °C using phase-contract microscopy and find that while we are able to engineer enzymes with significantly longer life-spans, increases in melting temperature do not correlate to increases in endurance at elevated temperature.

## Methods

### In silico design

Substitutions were selected based on Rosetta energy units as well as identifying substitutions displaying features associated with increased thermal stability, including increasing buried hydrophobic surface area [22], increasing quality of side-chain packing [23], increase surface charge of the tetrameric complex [24], decreasing conformational flexibility and improved packing at the symmetric dimer interface [25]. Computational approaches can be clustered into four topics:

1. Single substitutions were identified with in silico site saturation mutagenesis, using the Rosetta protein modeling software [21]. The in silico experiments used either a fixed-backbone approximation, where the backbone coordinates from the X-ray crystal structure were held fixed in the simulation, or a minimization of backbone and side-chain torsion angles prior to evaluating amino acid identities at each position.
2. Previous work comparing thermophilic and mesophilic enzymes showed that the backbone of structurally equivalent clusters can accommodate alternate sequence combinations by adjusting an average of 3.5 Å for each Cα atom [23]. Here, clusters of interacting residues were determined using a distance cutoff, where residues were considered interacting when any side-chain heavy-atoms were within 4 Å. Two clusters were identified for design targets based on poor side-chain packing, G6, I97, I166, A171 and H21, M42, Q44, Y46. These two clusters were designed by iterating between sequence design and minimization of backbone and side-chain torsion angles.
3. The PDC dimer displays symmetry at the interface resulting in a design constraint where any mutation made to one monomer must be mirrored in the bound partner. The symmetric design protocol simultaneously models the effects of an amino acid substitution at both positions at the interface [25].
4. Engineering a protein surface to have a high net charge can increase solubility and enhance thermal stability [24, 26]. Designing the PDC complex on the hypothesis that supercharging the surface can stabilize the interactions between domains, we identified substitutions to impart a high net charge while modeling the energetic consequences of the substitutions.

We selected PDC_1.01_ and PDC_1.10_ as the foundations for the second round of protein design based on the observed enhancements in thermal stability. We identified four additional mutations by selecting the next set of substitutions that would remove glycine residues (G491A, G515A, G516A, and G540A; Additional file 1: Table S1). We targeted the identification of additional glycine-removing mutations due to the successful thermal stabilization of PDC_1.01_, which contained two such mutations. These cases where a substitution removed a glycine residue underwent additional evaluation. Glycine, which has only a hydrogen atom at its side-chain position, can accommodate phi–psi backbone angles not accessible to any other natural amino acid. For this reason, any in silico mutations replacing a glycine residue underwent a filter based on Rosetta energy scores. The Rosetta energy term, p_aa_pp, gives the probability of finding an amino acid at given phi and psi dihedral angles. In silico mutations that removed a glycine residue were only considered if the p_aa_pp energy score was below 0.2. Additionally, glycine is the smallest amino acid. Mutations that passed the above filter were then sorted by the Lennard–Jones repulsive energy term (LJrep) to ensure significant atomic clashes were not present that might indicate the protein region could not accommodate the substitution (Additional file 1: Table S1). Glycine residues within 6 Å of the dimer active sites were not considered for redesign. We additionally avoided mutations introducing cysteine residues, as cysteine residues can participate in redox reactions [27].

A solvent exposed mutation in PDC_1.01_, A189K, was identified as a potentially beneficial single mutation in an in silico point mutation scan, but places a positively charged residue at the PDC surface and thus does not fit the goal of the negatively supercharged surface for PDC_1.10_. This mutation was subsequently removed in PDC_2.02_. A version of PDC_2.02_ that included the A189K mutation was characterized and evaluated using CD to confirm the mutation does not alter thermal stability based on observed changes in molar ellipticity (Additional file 1: Figure S3).

### Construction, cloning, and expression of PDC variants

DNA sequences for each PDC variant were codon optimized and cloned into a pET22b(+) vector (GenScript, Piscataway, NJ). The sequence for a hexahistidine tag was placed at the C terminus of the constructs. PDC protein variants were expressed either for 4 h at 37 °C or overnight at 25 °C with 0.1 mM IPTG in the BL21 (DE3) strain of *Escherichia coli*. Proteins were concentrated using Vivaspin spin columns with a molecular weight cutoff of 10,000 Da (GE Healthcare Life Sciences, Pittsburgh).

Protein purification. The frozen cell pellets were thawed at room temperature with equal volume of buffer A (50 mM Tris pH 7.5, 100 mM NaCl, 10 mM imidazole, 0.1 mM thiamine pyrophosphate (TPP), 0.5 mM dithiothreitol (DTT) and 1 mM MgCl_2_) and lysed with lysozyme and sonication. One mg/mL lysozyme (Hampton Research, Aliso Viejo, CA), 1.0 U/mL Pierce Universal Nuclease (Thermo Scientific, Rockford, IL) and EDTA-free protease inhibitor (Thermo Scientific, Rockford, IL) according to manufacturer instructions were added in the lysis mixture and incubated for 30 min at room temperature with occasional vortexing. Sonication was done at room temperature for 2 min using a Branson 5510 water bath sonicator (Branson Ultrasonics Corporation, Danbury, CT). Cell debris was removed by centrifugation at 15,000×*g* for 15 min. The supernatant was loaded onto an eight mL HisPur Cobalt column (Thermo Scientific, Rockford, IL) using an Akta FPLC system (GE Life Sciences, Piscataway, NJ) with buffer A (50 mM Tris pH 7.5, 100 mM NaCl, 20 mM imidazole, 0.1 mM TPP, 0.5 mM DTT, and 1 mM MgCl_2_). After loading and washing the unbound proteins from the column, PDC samples were eluted using 100% of Buffer B (50 mM Tris pH 7.5, 100 mM NaCl, 250 mM imidazole, 0.1 mM TPP, 0.5 mM DTT, and 1 mM MgCl_2_). Final purification was performed by size-exclusion chromatography using a HiLoad Superdex 200 (26/60) column (GE Healthcare, Piscataway, New Jersey, USA) in buffer C (20 mM Tris pH 7.5, 100 mM NaCl, 0.1 mM TPP, 0.5 mM DTT, and 1 mM MgCl_2_).

### Differential scanning calorimetry

Protein samples were analyzed by differential scanning calorimetry (DSC) using a Microcal VP-DSC instrument (Malvern, Worcestershire, UK) to measure the excess heat capacity of protein unfolding as a function of temperature. These measurements were used to directly calculate the enthalpy of unfolding (*∆H*_cal_) for each protein sample according to the equation:

$$\Delta H = \mathop \int \limits_{{T_{\text{f}} }}^{{T_{\text{u}} }} C_{\text{p}} {\text{d}}T$$

Protein samples were measured over a temperature range of 10–90 °C and at a scan rate of 60 °C/h. No feedback mode was used for each DSC experiment. Buffer baseline scans were established before each protein run by loading both the sample and reference cells with buffer and performing 3 or more heating and cooling cycles until the deviation in scans was less than 0.01 mcal/min. Buffer was then removed from the sample cell and replaced with the protein sample during the temperature range of 25–15 °C during the cool down cycle. All protein samples were diluted to 0.2 mg/mL in 20 mM TRIS pH 7.5, 100 mM MnCl, 1 mM MgCl_2_, 0.5 mM DTT, 0.1 mM TPP buffer and run at that concentration in order to minimize post-unfolding aggregation.

DSC data on each sample was analyzed by the Origin 7.0 software [28] coupled to a DSC data analysis module provided with the Microcal VP-DSC. Sample scans were buffer subtracted, normalized to molar concentration and corrected by baseline correction options in the DSC data analysis module prior to least squares analysis using a non-two-state model option. The fitted sample curves produced the melting temperature (*T*_m_), enthalpy of unfolding (*∆H*_cal_), and van’t Hoff enthalpy (*∆H*_vH_) for each transition.

### Circular dichroism spectroscopy and thermal melts

Circular dichroism (CD) spectra and thermal melt measurements were performed on a Chirascan-plus spectrometer (Leatherhead, Surrey, UK) using a 0.5-mm path-length quartz cuvette. WT and mutant PDC proteins were buffer exchanged into a potassium phosphate buffer (10 mM potassium phosphate, 100 mM NaCl, 1 mM MgCl_2_, 0.1 mM TPP, 0.5 mM DTT, pH 7.5) and prepared to a final concentration of 0.2 mg/mL. CD spectra were measured at the stated temperatures with a step size of 0.25 nm. The thermal melts were performed in continuous ramp mode at a rate of 2 °C/min while measuring CD at 222 nm. The *T*_m_ of each protein was determined from the first derivative of the thermal melt curves using Prism 6 (GraphPad, La Jolla, CA). Each thermal melt experiment was performed in triplicate.

### ThermoFluor high-throughput protein stability assay

The high-throughput ThermoFluor assay was used to evaluate the effects of the glycine-to-alanine substitutions G491A, G515A, G516A, and G540A. For the ThermoFluor assay, a hydrophobic dye, SYPRO Orange, binds to exposed hydrophobic regions of the protein. As proteins begin denaturation upon heating, increasing amounts of hydrophobic regions are exposed resulting in an increased signal. The assay is performed in an RT-PCR machine using 96-well plates, allowing the simultaneous characterization of many protein variants. This assay has been used to rapidly screen protein mutants generated from computational design approaches [29, 30], and thus allowed us to evaluate the threshold for selecting these mutations for combinatorial libraries.

SYPRO^®^ Orange Protein Gel Stain, supplied at 5000× concentrate in dimethyl sulfoxide (Thermo Scientific, Waltham, MA), was diluted to 200× in buffer (20 mM Tris pH 7.5, 100 mM NaCl, 0.1 mM TPP, 0.5 mM DTT, and 1 mM MgCl_2_). Wild-type PDC and variants were diluted to 5 μM in buffer, with protein concentrations determined by measuring absorbance at 280 nm. Extinction coefficients were calculated using the method described by Gill and von Hipple [31]. 50 μL samples were made by combining 45 μL of protein with 5 μL of 200× SYPRO^®^ Orange stain. Protein variants were measured in triplicate, with 50 μL samples placed in Hard-Shell^®^ 96-well PCR plates with clear wells (BioRad, Hercules, CA). Plates were covered with MicroAmp^®^ optical adhesive film (Thermo Scientific, Rockford, IL) to prevent sample evaporation. Spectra were obtained on a BioRad Real-Time C1000 Touch Thermal Cycler (BioRad, Hercules, CA). Thermal denaturations were done by increasing temperature from 25 to 95 °C at a rate of 1 °C/min, taking a plate read every minute using the FRET scan mode. Circular dichroism and ThermoFluor spectra were generated using IGOR Pro (WaveMetrics Inc., Lake Oswego, OR).

### PDC activity assay

PDC activity was measured using an assay where decarboxylation is coupled with alcohol dehydrogenase [32], and the conversion of NADH to NAD+ by the alcohol dehydrogenase was monitored for 5 min with a Varian Cary 400 (Agilent Technologies, Santa Clara, CA) temperature-controlled spectrophotometer at 25 °C. Four cuvettes, a blank and a triplicate of one sample of interest, were prepared and measured at a time. Each cuvette contained one mL of reaction mix and 20 µL of PDC with a suitable dilution in buffer D (20 mM BIS–Tris pH 6.5, 100 mM NaCl, 1 mM MgCl_2_, 0.1 mM TPP, 0.5 mM DTT), mixed using a 1 mL pipette (all samples in triplicate). Reaction mix contained 20 mM Na pyruvate (Fisher Scientific, Fair Lawn, New Jersey), 0.288 mM NADH (Sigma Chemical CO, St. Louis, MO) and 40 U yeast alcohol dehydrogenase (MP Biomedicals LLC, Solon, OH) in buffer D. Blank contained only the reaction mix. The Protein concentration was determined using the Bradford protein reagent with bovine serum albumin as the standard (BioRad, Hercules, CA).

### Crystallization

PDC_2.03_ crystals were initially obtained with sitting drop vapor diffusion using a 96-well plate with Grid Screen Salt HT from Hampton Research (Aliso Viejo, CA). Fifty microliter of well solution was added to the reservoir, and drops were made with 0.2 µL of well solution and 0.2 µL of protein solution using a Phoenix crystallization robot (Art Robbins Instruments, Sunnyvale, CA). The crystals were grown in 0.1 M MES monohydrate pH 6.0 and 2.4 M ammonium sulfate at 20 °C. The protein solutions contained 6 mg/mL of protein in 20 mM Tris pH 7.5, 100 mM NaCl, 1 mM MgCl_2_, 0.5 mM DTT, and 0.1 mM TPP.

### Data collection and processing

The PDC_2.03_ crystals were flash frozen in a nitrogen gas stream at 100 K before home source data collection using an in-house Bruker X8 MicroStar X-ray generator with Helios mirrors and Bruker Platinum 135 CCD detector. Data were indexed and processed with the Bruker Suite of programs version 2014.9 (Bruker AXS, Madison, WI).

### Structure solution and refinement

Intensities were converted into structure factors, and 5% of the reflections were flagged for Rfree calculations using programs F2MTZ, Truncate, CAD, and Unique from the CCP4 package of programs [33]. The program MOLREP [34] version 11.4.06 was used for molecular replacement using wild-type PDC (PDB code 2WVG [35]) as the search model. Refinement and manual correction was performed using REFMAC5 [36] version 5.8.0155, and Coot [37] version 0.8.6. The MOLPROBITY method [38] was used to analyze the Ramachandran plot, and root-mean-square deviations (RMSD) of bond lengths and angles were calculated from ideal values of Engh and Huber stereo chemical parameters 47 [39]. Wilson B-factor was calculated using CTRUNCATE version 1.15.10 [33]. The data collection and refinement statistics are shown in Additional file 1: Table S2.

### Structure analysis

**Fig. 2:**
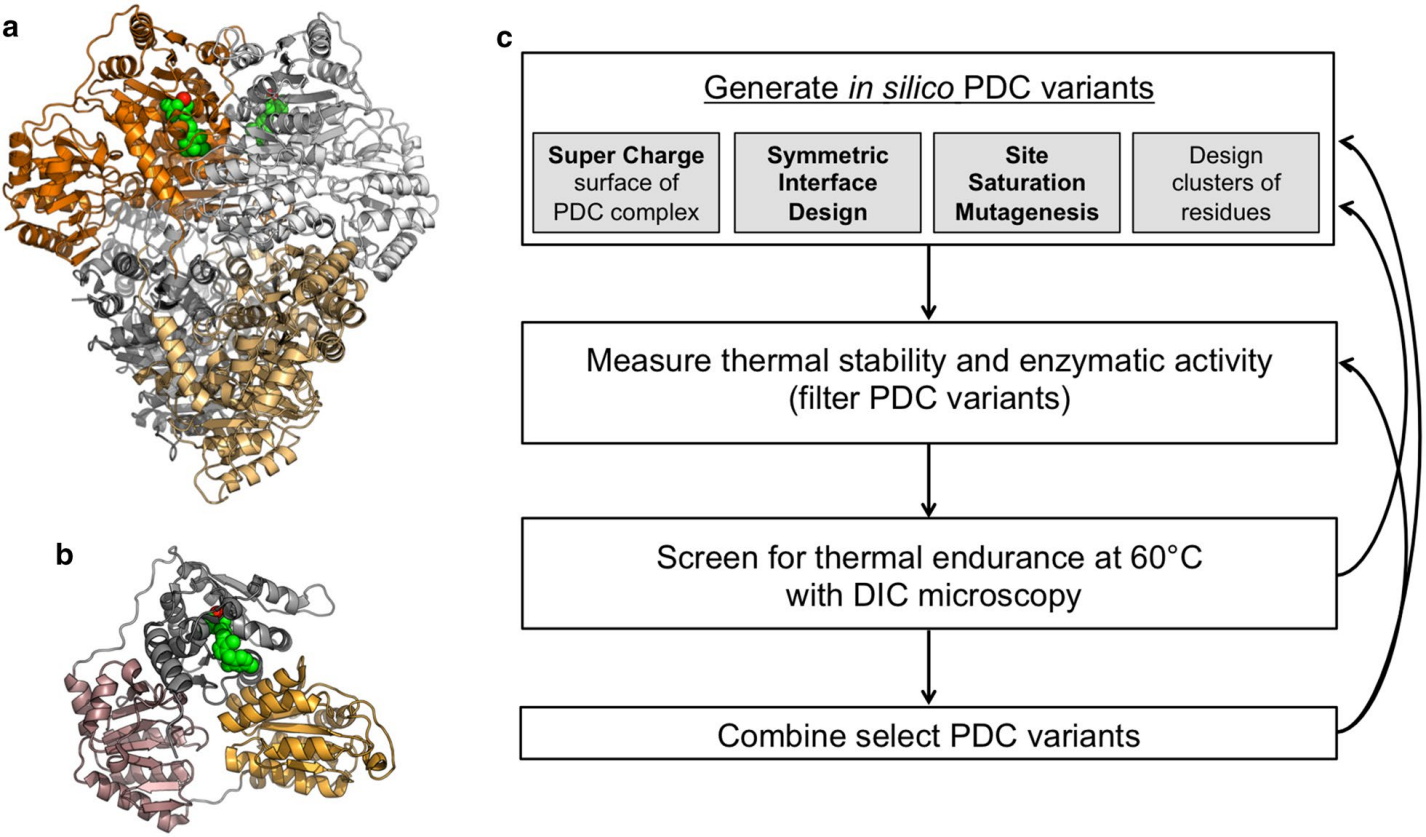
A thermostable PDC with ability to resist denaturation and maintain an active complex. **a** PDC with chains A and E represented in dark and light orange, respectively, and chains B and F in white and gray, respectively (PDB code 2WVA). The cofactors located at the active sites between chains A and B, the dimer interface, are shown as spheres, with thiamine diphosphate (TPP) in green and magnesium (Mg^2+^) in red. **b** The PDC monomeric unit composed of three independent structural regions, shown in gray, orange, and pink. TPP is shown in green and Mg^2+^ is shown in red. **c** An interactive computational design and experimental characterization workflow allows for the accumulation of thermostabilizing mutations throughout the homotetrameric PDC.

Programs Coot 45, PyMOL (http://www.pymol.org) and ICM (http://www.molsoft.com) were used for comparing and analyzing structures. Figures 2a, b, 5 were created using PyMOL. The root-mean-square deviation (RMSD) between the monomeric unit of PDC and PDC_2.03_ was computed using PyMol (The PyMOL MolecularGraphics System, Version 1.5.0.4 Schrödinger, LLC.)

### DIC microscopy

Pyruvate decarboxylase (PDC) protein preps were diluted in buffer to 1 mg/mL concentration. 7.5 µL of protein solution was placed between two glass coverslips separated by a 0.15 mm deep SecureSeal imaging spacer (Grace Bio-labs, Bend, OR). Heating was controlled using a Linkam FTIR600 temperature-controlled microscope stage (Linkam Scientific Instruments, UK) and heated from 24 °C to either 60 or 120 °C at a ramp rate of 1 °C/min. The optics were set up for bright field differential interference contrast imaging on a NikonE800 microscope (Nikon, Tokyo, Japan), using a 20× 0.75 NA PlanApo ELWD objective. Images were captured every 30 s over the 3 h using a SPOT RTKE CCD camera (Diagnostic Instruments, Sterling Heights, MI) as TIFF stacks. TIFF stacks were analyzed using FIJI (ImageJ).

## Results

### Computational design of a thermostable PDC

PDC self-associates to form a 260 kDa dimer of dimers, with two active sites buried at each dimer interface (Fig. 2a) [35]. A thermostable and active PDC will likely require stabilization of the monomer to resist denaturation at high temperatures and also stabilization of the interactions between the monomers to maintain the active complex. In addition, the monomeric unit of PDC is composed of three distinct structural regions (Fig. 2b). Our protein-engineering goal was to identify thermostabilizing mutations located in each structural region to address the distinct components and interactions of the PDC complex.

We used multiple design strategies to identify amino acid substitutions distributed throughout the PDC structure (Table 1). First, single substitutions were identified with in silico site saturation mutagenesis, combining mutations to decrease the number of variants selected for experimental characterization (PDC_1.01_ through PDC_1.04_). Next, clusters of spatially interacting residues were redesigned simultaneously to improve regions with poor atomic packing, iterating between backbone minimization and amino acid design (PDC_1.05_ and PDC_1.06_). Additionally, the PDC dimer displays symmetry at the interface resulting in a design constraint where any mutation made to one monomer must be mirrored in the bound partner. A symmetric interface design protocol simultaneously models the effects of an amino acid substitution at both positions at the interface (PDC_1.07_ and PDC_1.08_) [25]. Lastly, engineering a protein surface to have a high net charge can increase solubility and enhance thermal stability [24, 26]. Designing the PDC complex on the hypothesis that supercharging the surface can stabilize the interactions between domains, we identified substitutions to impart a high net charge while modeling the energetic consequences of the substitutions (net positive charge for PDC_1.09_ and net negative charge for PDC_1.10_) [40].

**Table 1:** Designing thermostabilizing mutations for the PDC homotetrameric complex

PDC variant	Mutations	Activity at 25 °C
1.01	G224A, V374I, G540A	Yes
1.02	F55W, G224A, V374I	Yes
1.03	F55W, G224A, V374I, G540A	Yes
1.04	G224A, G540A	Yes
1.05	G6A, I97V, I166F, A171F	ND^a^
1.06	H21I, M42F, Q44V, Y46F	Yes
1.07	A77V, L78F, S79M, A80M, A83V, I84V, G85V, G86M, M127V, A128M, Y163F	ND^a^
1.08	A77V, L78F, S79M, A80M, D82V, A83V, I84V, G85V, G86M, M127V, A128M	ND^a^
1.09	S2R, N41K, A207K, Q333K, A357K, T379R, N402K, V524K, A527K, A557K	ND^a^
1.10	S2D, L38D, A189K, A207E, Q333E, A357D, A376D, A519E, A527D, K553E	Yes
2.01	G109A, G224A, V374I, G491A, G515A, G516A, G540A	Yes
2.02	S2D, L38D, A207E, G224A, Q333E, A357D, V374I, A376D, A519E, A527D, G540A, K553E	Yes
2.03	S2D, L38D, G109A, A207E, G224A, Q333E, A357D, V374I, A376D, G491A, G515A, G516A, A519E, A527D, K553E, G540A	Yes

PDC variants were expressed in *E. coli* and evaluated for activity

^a^ND. The activity for these designs was not determined as they did not express on the first attempt or expressed in such a small amount that not enough protein was obtained to evaluate the activity or thermal stability

The mutations display features associated with increased thermal stability, including increased buried hydrophobic surface area [22], enhanced quality of side-chain packing [23], increased surface charge of the tetrameric complex [24], decreased conformational flexibility and improved packing at the symmetric dimer interface [25]. A summary of the designs is shown in Table 1. Our approach utilizes an iterative workflow, where the most promising designs are selected after experimental characterization, then combined, and/or further enhanced with additional mutations (Fig. 2c). The iterative computational design approach is key to identifying thermostabilizing mutations that work together to stabilize any structurally weak regions.

### Identification of PDC designs with enhanced thermostability

To evaluate whether the PDC designs resulted in an enhancement in thermal stability, genes encoding wild-type PDC and variants listed in Table 1 were expressed in *Escherichia coli*. Enzymatic activity for all PDC enzymes was determined, with all successfully expressed PDC enzymes showing similar levels of activity. Pyruvate decarboxylation is measured by coupling PDC to an NADH-dependent alcohol dehydrogenase (ADH). The oxidation of NADH by ADH upon the conversion of acetaldehyde to ethanol results in a colorimetric change. NADH is not stable at elevated temperatures, thus activity was determined at 25 °C [41]. Any designs that did not produce a detectable level of soluble protein in the first attempt were discarded, and activity for these designs was labeled “ND”, or not determined, in Table 1.

Differential scanning calorimetry (DSC) was initially used to evaluate the thermal stability of the PDC variants. DSC scans displayed multiple peaks, possibly due to the multimeric attribute of PDC, challenging the comparison of some PDC variants (Additional file 1: Figure S2). DSC results served to eliminate a few underperforming designs (PDC_1.02_, PDC_1.04_, PDC_1.03_, PDC_1.07_), but we instead relied on circular dichroism (CD) to characterize the remaining variants. PDC was shown to have irreversible unfolding, thus the melting temperatures reported here for PDC variants indicate relative thermal stabilities.

**Fig. 3:**
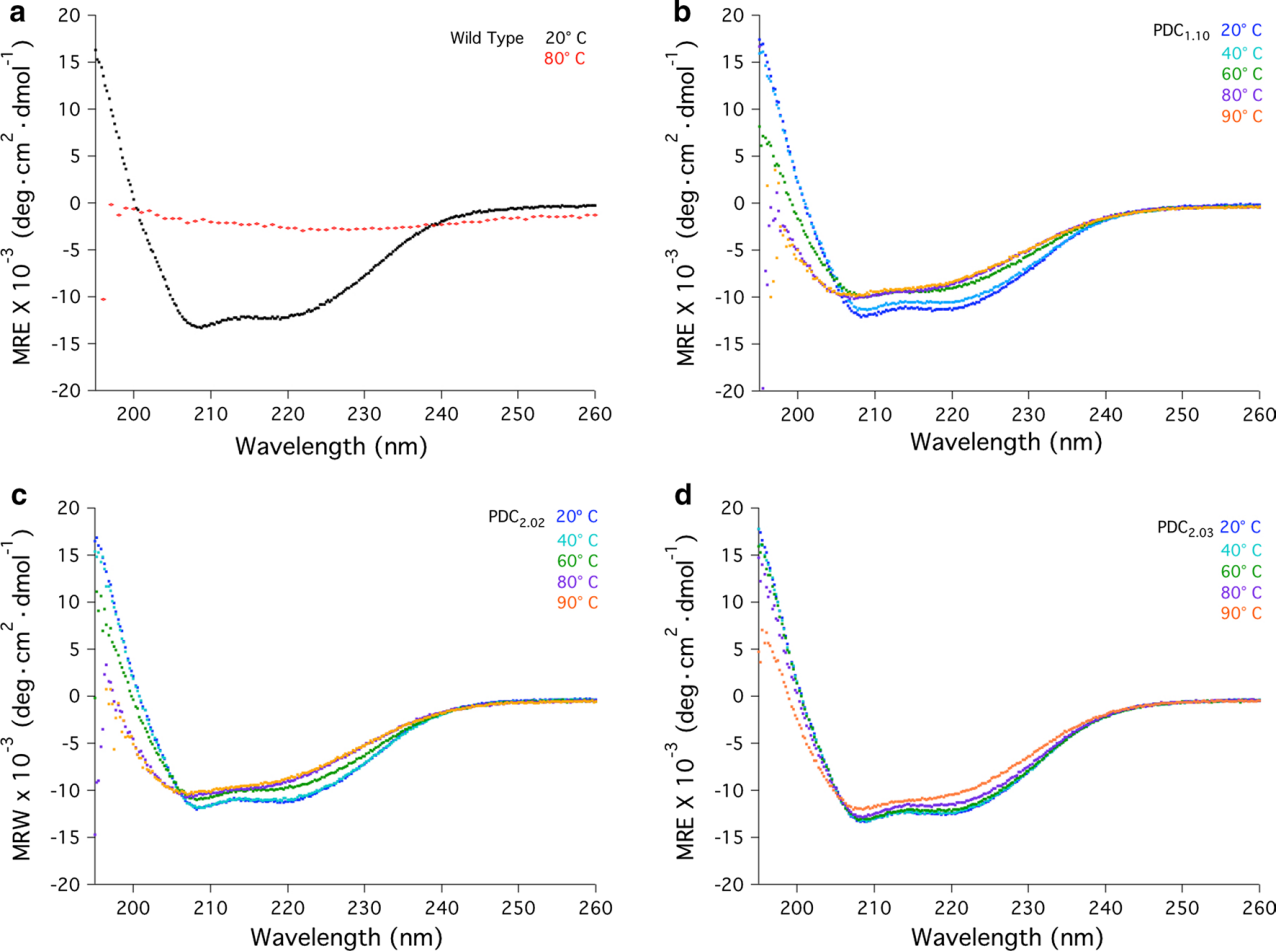
Evaluation of the thermal stability of PDC designs using molar ellipticity, with the temperature being increased from 20 to 90 °C. **a** Comparing the molar ellipticity for wild-type PDC at 20 °C and upon complete thermal denaturation by 80 °C. Molar ellipticity measurements are shown for designs **b** PDC_1.10_, **c** PDC_2.02_, and **d** PDC_2.03_, heating from 20 to 90 °C

Two PDC variants were selected for additional characterization from the first round of design. Evaluating ellipticity at 222 nm PDC_1.01_ displayed an increased *T*_m_ of 4 °C relative to wild type, with melting temperatures of 66 and 62 °C, respectively (Additional file 1: Figure S1). PDC_1.10_, interestingly, did not undergo a large change in ellipticity at 222 nm, indicating the protein did not fully denature or precipitate by 90 °C. We therefore assessed the thermal stability of PDC_1.10_ by measuring molar ellipticity from 195 to 260 nm to evaluate changes in a broader range of secondary structural elements (Fig. 3b). For comparison, wild-type PDC was evaluated from 195 to 260 nm at 20 °C and also at 80 °C, the temperature by which PDC is fully denatured (Fig. 3a). Unlike the wild-type PDC, PDC_1.10_ resists thermal denaturation up to 90 °C. However, loss of some secondary structure indicated by a loss of molar ellipticity is observed at 60 °C.

Three tiers of design were attempted for the second round of PDC engineering. First, PDC_1.01_ was further improved by the addition of four substitutions, generating PDC_2.01_. Second, PDC_1.01_ and PDC_1.10_ were combined to evaluate the additivity of these two successful designs, generating PDC_2.02_. In the third design, the same four substitutions were combined with PDC_1.01_ and PDC_1.10_, generating PDC_2.03_. The third approach was the most thorough test of both the accuracy of our computational predictions and the combinability of the designs.

The thermal stability of PDC_2.01_, containing the four substitutions and the parent design PDC_1.01_, was evaluated using the high-throughput ThermoFluor assay (Additional file 1: Figure S1B, A, respectively). PDC_2.01_ exhibited a melting temperature nearly identical to PDC_1.01_, displaying no improvement in thermal stability. Nearly all of the pairwise combinations displayed a *T*_m_ within one or two degrees of the parent PDC_1.01_. The most significant increase in *T*_m_ was seen with the addition of G491A and G516A, resulting in an increase of 4 °C.

PDC_2.02_ and PDC_2.03_ were evaluated by measuring molar ellipticity from 195 to 260 nm, as done with the parent design, PDC_1.10_ (Fig. 3c). PDC_2.02_ resists thermal denaturation, as seen with PDC_1.01_. PDC_2.02_ also loses some secondary structure at 60 °C, as seen with PDC_1.01_. Thus, while PDC_2.02_ does not fully denature up to 90 °C, it also does not display an apparent increase in thermal stability compared to PDC_1.01_ based on changes in molar ellipticity.

A significant increase in thermal stability is observed with PDC_2.03_, which combines PDC_1.01_, PDC_1.10_ and the additional four substitutions. PDC_2.03_ does not show loss of secondary structure until 90 °C, well above the target growth temperature of 60 °C, at which point small changes in molar ellipticity are observed (Fig. 3d). Importantly, PDC_2.03_ differs from PDC_2.02_ by only four substitutions. The combined ThermoFluor and CD results indicate, therefore, the same four mutations have no apparent thermostabilizing effect in the context of PDC_2.01_ while significantly increasing thermal stability in the context of PDC_2.03_.

### Measuring the structural endurance of PDC variants

The aim of this work was to develop an iterative computational protein design protocol to rationally engineer a mesophilic enzyme, PDC from *Z. mobilis*, to function like a thermophilic enzyme. The maximum growth temperature for *Z. mobilis* is 42 °C [20], well below the optimum growth temperature of 60 °C for *C. thermocellum* [19]. While our target temperature of 60 °C is below the *T*_m_ of wild-type PDC (Additional file 1: Figure S1), where half the protein has been thermally inactivated, changes in ellipticity indicate PDC is at or approaching a structurally unstable temperature.

Seeking an approach capable of making time-resolved measurements of PDC variants at the target temperature of 60 °C, we evaluated variants using differential interference contrast (DIC) microscopy during a heat and hold experiment. This approach is similar to optical density and static light scattering experiments, where blockage or scattering of light indicates changes in the size of protein species, generally due to aggregation [42, 43]. In the approach used here to evaluate the structural endurance of PDC variants, we combined measured changes in birefringence with precise temperature control to evaluate protein aggregation upon thermal denaturation.

**Fig. 4:**
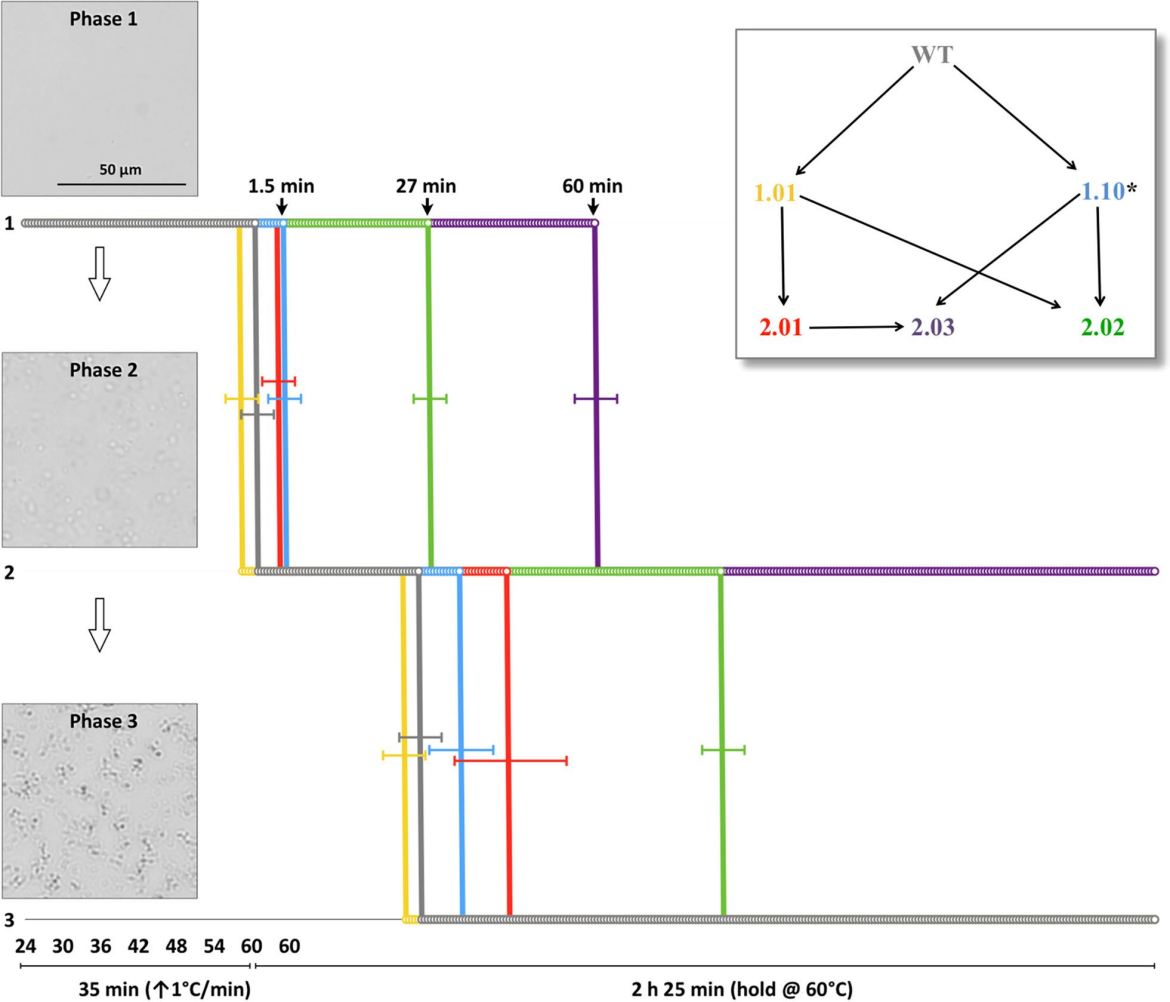
Real-time differential interference contrast (DIC) microscopy of PDC variants showing three distinct phases. PDC variants were evaluated during a heating ramp (1 °C/min) from room temperature to 60 °C followed by a temperature hold at 60 °C. In phase 1, the protein is in solution with no visible birefringence. In phase 2, the protein becomes visible, forming an alternative conformation that appears to be soluble as it is still moving in solution. In phase 3, all of the protein has aggregated into a semicontinuous lattice of precipitated protein. The graph displays the temperature at which each of the PDC variant transitions between phases. Wild-type PDC (WT) is shown in gray, PDC_1.01_ in yellow, PDC_1.10_ in blue, PDC_2.01_ in red, PDC_2.02_ in green, and PDC_2.03_ in purple. PDC_1.01_ (yellow) and PDC_1.10_ (blue) were combined to generate PDC_2.02_ (green). PDC_2.01_ (red) and PDC_1.10_ (blue) were combined to generate PDC_2.03_ (purple). During the 60 °C experiments, PDC_2.03_ protein never formed a lattice of precipitated protein to enter phase 3. *Variant PDC_1.10_ includes the mutation A189K. Variant 2.02 was evaluated with and without the mutation A189K (see Additional file 1: Figure S3 and Fig. 3c, respectively). Thermal stability was the same with and without the mutation, and thus A189K was not included in variant 2.03

The proteins were heated from 25 °C at a ramp rate of 1 °C/min to a final temperature of 60 °C and then held at 60 °C for a total time of 3 h. Using DIC analysis we observe three distinct phases of protein behavior. In the starting condition (phase 1), the protein is in solution and invisible to DIC imaging, producing no birefringent margins. As the protein is heated, it undergoes a structural change, forming an alternate but stable conformation, (phase 2) and becomes visible. This alternate conformation appears to be soluble, as the phase 2 structures are observed moving in solution. Finally, all of the protein appears to aggregate into a semicontinuous lattice of precipitated protein (phase 3). Representative micrographs of these three phases are shown at the left side of Fig. 4. These observed structural phases fit the model proposed by Tomazic and Klibanov, where soluble proteins (phase 1) can undergo a partial unfolding into kinetically stable structures (phase 2) followed by the irreversible formation of aggregates (phase 3) [44, 45].

Wild-type PDC entered phase 2 immediately upon reaching 60 °C, remaining in phase 2 for approximately 25 min before reaching phase 3. PDC_1.01_, which exhibited a four degree increase in *T*_m_ based on ellipticity measurements, showed no improvement over wild-type PDC when held at 60 °C. PDC_1.10_, which resisted denaturation and precipitation in CD experiments to at least 90 °C, similarly showed little to no improvement over wild-type PDC when held at 60 °C. PDC_1.10_ results suggest that the loss of molar ellipticity observed at 60 °C in CD experiments indicates a structural instability and does not represent a stable conformation.

All second round designs (PDC_2.01_, PDC_2.02_ and PDC_2.03_) outperform the first round designs, (PDC_1.01_ and PDC_1.10_). PDC_2.01_ enters phase 2 within minutes after reaching 60 °C but remains in phase 2 for approximately 35 additional minutes before entering phase 3. PDC_2.02_ and PDC_1.10_, which displayed nearly identical changes in molar ellipticity, have markedly different behavior when monitored at 60 °C. PDC_2.02_ remains in phase 1 for 27 min at 60 °C before entering phase 2. PDC_2.02_ then lasts another 90 min before entering phase 3, outperforming the parent designs PDC_1.01_ and PDC_1.10_.

PDC_2.03_, which displayed no loss of molar ellipticity until 90 °C when monitored by CD, lasted 60 min at 60 °C before entering phase 2, and never entered phase 3 for the duration of the experiment. Thus, the four additional mutations (G491A, G515A, G516A, and G540A) displayed manifestly different stabilizing effects depending on whether they were evaluated in the context of design PDC_2.01_ or PDC_2.03_.

### Structural evaluation of PDC_2.03_

**Fig. 5:**
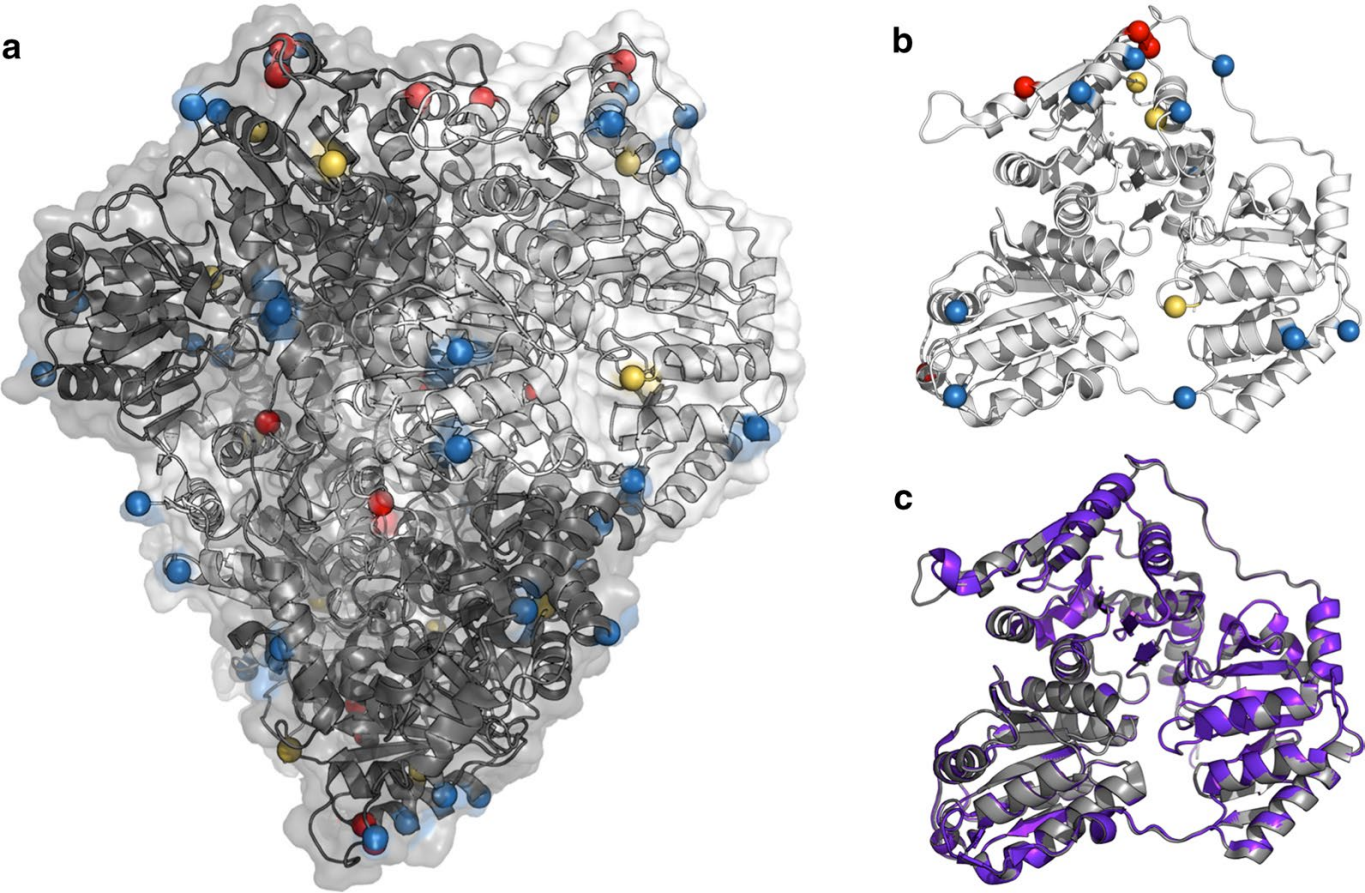
Mutations in PDC_2.03_ imparting an increase in thermal stability and endurance without changing the backbone. **a** The Cα atoms at mutated positions are highlighted as spheres. The mutations that comprise PDC_2.03_ are located throughout the PDC enzyme. Mutations from PDC_1.01_ are shown in yellow, PDC_1.10_ in blue, and the mutations added to generate PDC_2.03_ are shown in red. Chains A and E are shown in dark gray, and chains B and F are shown in light gray, respectively. **b** The mutations in PDC_2.03_ are found in each of the three structural regions of PDC monomer. **c** The X-ray crystal structure of PDC_2.03_ (PDB code 5TMA) is shown in purple aligned with the wild-type PDC in gray (PDB code 2WVA). The Cα backbone root-mean-square deviation (RMSD) for the PDC monomeric unit is 0.19 Å

PDC_2.03_ contains sixteen mutations in the monomeric unit and sixty-four mutations in the homotetrameric complex (Fig. 5a). The mutations are found throughout the PDC complex as well as in each of the three structural regions of the monomeric unit (Fig. 5b). The wild-type PDC has a *T*_m_ of 62 °C, while PDC_2.03_ resists thermal denaturation to at least 90 °C. We used several approaches to identify potential thermostabilizing mutations, but the mutations included in PDC_2.03_ were all generated using a fixed-backbone approach, where the backbone coordinates were held fixed to the dihedral angles observed in the crystal structure (PDB code 2WVA).

To determine whether PDC_2.03_ retained the backbone conformation from the design runs, we elucidated the X-ray crystal structure of the PDC variant (PDB code 5TMA). The structure of PDC_2.03_ was refined to a resolution of 1.67 Å with R and R_free_ of 0.170 and 0.203, respectively. There are two molecules in the asymmetric unit in complex with TPP and magnesium. Each monomer has three domains with an open α/β topology [46] with several ethylene glycol and sulfate molecules on the surface.

The backbone root-mean-square deviation (RMSD) between chain A of the wild-type PDB and PDC_2.03_ is 0.19 Å (Fig. 5c). The largest conformational difference, located in the upper left of Fig. 5c, is located at a crystal contact point and distant from both the active sites and any of the 16 mutations. Thus, the mutations impart significant enhancement in thermal stability without unintended structural alterations.

## Conclusions

In this study, we used iterative computational protein design to rationally engineer the mesophilic enzyme, pyruvate decarboxylase, to exhibit enhanced thermal endurance at the growth temperature of a thermophilic organism. The target temperature of 60 °C is below the *T*_m_ of the wild-type PDC, at 62 °C, but is in a thermally unstable region as determined by changes in ellipticity (Additional file 1: Figure S1A). We identified a series of thermostabilizing mutations, increasing the *T*_m_ of PDC by 4 °C in one case (PDC_1.01_) and generating a variant that resists thermal denaturation to at least 90 °C (PDC_1.10_) in another. While increases in structural endurance at elevated temperature are not achieved by any single design, the combination of designs resulted in a PDC variant with a large increase in thermal endurance at 60 °C.

We find, however, that increases in *T*_m_ do not directly correlate with enhanced thermal endurance. Designs PDC_1.10_ and PDC_2.02_, for example, exhibit no observable difference in thermal stability based on measured changes in molar ellipticity, but display strikingly different thermal endurance; PDC_2.02_ survives approximately twice as long at 60 °C compared to PDC_1.10_. Similarly, PDC_1.10_ shows significant thermal stabilization compared to wild-type PDC, resisting thermal denaturation to at least 90 °C, yet survives only minutes longer at 60 °C.

The explanation for these observed incongruities may have to do with cooperativity between the mutations. Fersht introduced the concept of a double-mutant cycle to determine whether two residues are energetically coupled, either through a direct or indirect interaction [47]. Based on this analysis, two residues are coupled if the change in free energy for the double mutant is not equal to the sum of the changes in free energy for each mutation individually. This concept has been expanded to include changes in free energy associated with structural or functional properties upon a double mutation [48]. Here we have generated PDC variants which all included multiple mutations. However, the energetic nonadditivity expected between mutations of coupled residues is clearly observed when comparing the thermal endurance of PDC variants (Fig. 4). PDC_2.02_, which resulted from the combination of PDC_1.01_ and PDC_1.10_, displays an increase in structural endurance that far exceeds what would be achieved if the mutations were additive. Similarly, PDC_2.03_ survives much longer at 60 °C than would be expected if the residues in the parent designs, PDC_1.10_ and PDC_2.01_, were not energetically coupled. None of the sixteen mutations that comprise PDC_2.03_ are directly interacting and are generally structurally removed from one another. Thus, the observed energetic coupling is likely due to structural stabilizations in interacting regions of the PDC.

Further, the glycine-to-alanine mutations (G109A, G491A, G515A, and G516A) included in PDC_2.01_ and PDC_2.03_ display context-dependent behavior. These four mutations do not increase thermal stability when added to PDC_1.01_ to generate PDC_2.01_, and similarly do not result in a significant increase in thermal endurance. However, these same four mutations increase both thermal stability and thermal endurance when added to PDC_2.02_ to generate PDC_2.03_. On the one hand, these results suggest that the high-throughput thermal stability screens may fail to identify thermal stabilizing mutations if they are not evaluated in the favorable context of a more thermostable protein. However, the context dependence of these mutations also demonstrates the importance of cooperativity, such that even mutations that by themselves yield only the smallest gains in stability can ultimately make a significant contribution when part of a larger design.

These results suggest stabilizing all regions of a protein at or above the target temperature is critical to achieve the desired enhancement in thermal endurance. PDC_1.10_ and PDC_2.02_ both exhibit some loss of molar ellipticity at 60 °C, the temperature at which we evaluate thermal endurance by DIC. The improvement in endurance for PDC_2.02_ compared to PDC_1.10_, which is not observed as an improvement in stability based on the thermal melt, may therefore be the result of structural stabilization near 60 °C. In addition, PDC_2.03_ exhibits the highest degree of thermal endurance at 60 °C and also shows no loss of molar ellipticity until 90 °C. Both the context dependence of the four glycine-to-alanine mutations as well as the pronounced cooperativity observed in the combined mutations of PDC_2.03_ may be a reflection of the thermal stabilization of structurally cooperative regions of the multimeric PDC.

Much of protein design and structure prediction is predicated on the hypothesis that the native structure for a given protein is the conformation with the lowest Gibbs free energy. However, there is significant evidence that for many proteins the active and native conformation may not be the lowest energy conformation, but instead kinetic energy barriers drive the folding trajectories to achieve the observed native conformations [18]. For proteins that fall into such kinetically driven folding trajectories, perturbing physiological conditions such as temperature or pH can alter protein folding and subsequently alter the sampled conformations. Thus, a critical next step and test of our ability to computationally design thermostable enzymes is to evaluate protein folding under elevated temperatures.

## Additional file

**Additional file 1.** Additional figures and tables.
