# Supplementary material for: An iterative computational design approach to increase the thermal endurance of a mesophilic enzyme
Source: Biotechnol Biofuels. 2018 Jul 9;11:189. doi: 10.1186/s13068-018-1178-9 (PMC6036693; doi:10.1186/s13068-018-1178-9)
Supplement: Supplementary file 1 — Additional file 1. Additional figures and tables. [file 13068_2018_1178_MOESM1_ESM.docx]

Additioanl Information

**Figures**

**Figure S1.** Thermal melts showing (**a**) circular dichroism spectra for wild type PDC compared to design PDC_1.01_ and (**b**) showing the high-throughput ThermoFluor assay to measure the thermal stability of PDC_1.01_ and PDC_2.01_. Four mutations were added to PDC_1.01_ to generate PDC_2.01_. These four mutations are listed as A (G109A), B (G491A), C (G515A), and D (G516A), and a pair-wise analysis of these mutations against the PDC_1.01_ design are evaluated and compared.

**Figure S2.** Differential Scanning Calorimetry was initially used to eliminate PDC variants. DSC scans are shown for (**a**) wild type PDC, (**b**) PDC_1.01_ and (**c**) PDC_1.03_.

**Figure S3.** Evaluating the thermal stability of PDC_2.02_ with an additional mutation, A189K, from one of the parents PDC variants, PDC_1.10_. The thermal stability is evaluated by measuring molar ellipticity from 195 to 260 nm, heating from 20 °C to 90 °C. The A189K mutation did not fit the goal for PDC_2.02_ of designing a negatively charged surface, and the mutation was therefore removed.

**Table S1**. Designing mutations to decrease conformational flexibility of the PDC monomer.

Mutations selected for experimental characterization are shown in bold and underlined. All positions with a glycine as the wild type residue were considered for redesign. Energies are sorted by Lennard Jones repulsive energy (LJ_rep_). Mutations were selected for experimental characterization if p_aa_pp >0.2 or LJ_rep_ >10.

**Table S2.** X-ray data collection and refinement statistics. Statistics for the highest resolution bin are in parenthesis.

| Data collection | | |
| --- | --- | --- |
| Space group | P 4_3_2_1_2 |  |
| Unit cell, Å, ° | a= b= 124.44, c = 173.83  α= β = γ= 90.0 |  |
| Wavelength, Å | 1.54188 |  |
| Temperature (K) | 100 |  |
| Resolution, Å | 50.0-1.67 (1.77–1.67) |  |
| Unique reflections | 157603 (24868) |  |
| R_int_^†^ | 0.072 (0.787) |  |
| Average redundancy | 10.4 (5.0) |  |
| <I>/<σ(I)> | 16.6 (1.1) |  |
| Completeness, % | 99.8 (98.6) |  |
| Refinement | | |
| Resolution, Å | 50-1.67 (1.71-1.67) |  |
| R/R_free_ | 0.170 (0.363)/ 0.203 (0.364) |  |
| Protein atoms | 9002 |  |
| Water molecules | 1028 |  |
| Other atoms | 225 |  |
| RMSD from ideal bond length, Å^#^ | 0.021 |  |
| RMSD from ideal bond angles, ° ^#^ | 1.949 |  |
| Wilson B-factor | 19.6 |  |
| Average B-factor for protein atoms, Å^2^ | 24.1 |  |
| Average B-factor for water molecules, Å^2^ | 33.0 |  |
| Ramachandran plot statistics, %* |  |  |
| Allowed | 99.8% |  |
| Favored | 97.6% |  |
| Outliers | 3 |  |

† R_int_ = ∑| I - <I> | / ∑|I| where I is the intensity of an individual reflection and <I> is the mean intensity of a group of equivalents and the sums are calculated over all reflections with more than one equivalent measured

# [48], * [38]
